# The complete mitochondrial genome of *Holothuria fuscocinerea* (Jaeger,1833)

**DOI:** 10.1080/23802359.2019.1691950

**Published:** 2019-12-09

**Authors:** Yi Sun, Beichen Ding, Chao Guo, Rui Han, Junhao Li, Feixiang Rong, Jun Ding

**Affiliations:** Key Laboratory of Mariculture and Stock Enhancement in North China's Sea, Ministry of Agriculture and Rural Affairs, Dalian Ocean University, Dalian, 116023, P. R. China

**Keywords:** Mitochondrial genome, sea cucumber, *Holothuria fuscocinerea*

## Abstract

In this study, the complete mitochondrial genome of *Holothuria fuscocinerea* was sequenced on an Illumina platform and assembled using NovoPlasty v. 2.7.1. It was submitted to NCBI GenBank and is available with accession number MN542416. The genome was 15,827 bp in size and contains 22 tRNA genes, 12 protein-coding genes, and 2 rRNA genes. The composition of A + T in *Holothura spinifera* mtDNA was 60.30%. Except ND6 and 5 tRNAs, the others are not on the H-strand. The phylogenetic relationship of 13 species of sea cucumber were analyzed using the neighbor-joining method by software MEGA5.0. *Holothuria fuscocinerea* was most closely related to *Holothuria polii*.

*Holothuria fuscocinerea* belong to the Family of holothuriidae, *Holothuria fuscocinere*a, being a benthic creature that feeds on debris and sediment species, it lives in the depth of 0–30 m ocean (http://www.marinespecies.org/aphia.php?p=taxdetails&id=210873), It usually lives in Celebes and Amboina; Ceylon, Bay of Bengal, East Indies, north Australia, Philippine, China, south Japan and South Pacific, Australia. By comparing with other mitochondrial genomes of sea cucumber, It provides a scientific basis for the systematic evolution of sea cucumber. Therefore, it is very important to study the complete mitochondrial genome of *Holothuria fuscocinerea*.

Samples used for sequencing were collected in Sanya, Hainan province, China (18°37′N 109°23′E). Keep it at −80 °C before sequencing. We isolated the complete mitochondrial genome of *Holothuria fuscocinerea* and sequenced it based on the Illumina pair-end technology. Assembled using SOAP denovo V2.04 software (http://soap.genomics.org.cn/). We forecast gene by using DOGMA software (http://dogma.ccbb.utexas.edu/), The genome contains rRNA and tRNA. MEGA5.0 (Kumar et al. 2016; Saitou and Nei 1987; Tamura et al. 2004) was put to use for multiple alignment. The mtDNA map of *Holothura spinifera* mitochondrial genome was drew with the online tool OGDraw (https://chlorobox.mpimp-golm.mpg.de/OGDraw.html) (Lohse et al. 2013). The specimen is stored in the Key Laboratory of Mariculture and Stock Enhancement in North China's Sea, Ministry of Agriculture and Rural Affrairs, Dalian Ocean University (voucher number: DLOU-KLM-SC01).

The size of the complete mitochondrial genome of *Holothuria fuscocinerea* is 15,827 bp (GenBank registration number: MN542416), including 12 protein-coding genes, 22 tRNA genes, and 2 rRNA genes. The mitochondrial genome of *Holothuria fuscocinerea* composition is 33.04% for A, 23.88% for C, 15.82% for G, and 27.25% for T. The percentage of A + T in *Holothuria fuscocinerea* mtDNA was 60.30%. Except ND6 and 5 tRNAs (tRNA-Gln, tRNA-Asp, tRNA-Val, tRNA-Ala, tRNA-Ser), the others are not on the H-strand. All protein initiation codons are ATG, except for ND4L. The termination codon of most protein-coding genes was TAA(6 of 12 genes) or TAG (2 of 12 genes). The 22 tRNA coding genes ranged in size from 60 bp to 76 bp.

We analyzed the phylogenetic relationship of 13 species of sea cucumber using the Neighbor-Joining method by MEGA5.0. The results revealed that the *Holothuria fuscocinerea* were most affinitive to the *Holothuria polii*. And the distance between *Holothuria forskali* and *Holothuria fuscocinerea* was the farthest (Figure 1). The results of the study help to construct the molecular identification system of the sea cucumber family and contribute to the phylogenetic research of the sea cucumber family.

**Figure 1.**
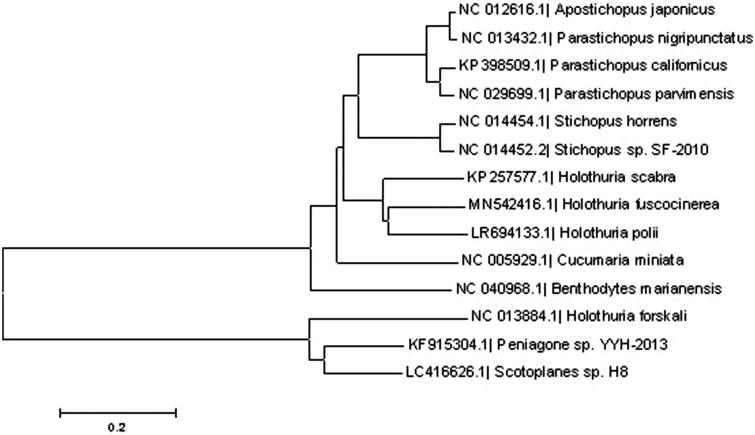
Consensus neighbor-joining tree based on the complete mitochondrial sequence of *Holothuria fuscocinerea* and other 13 species of sea cucumber. The phylogenetic tree was constructed using MEGA 5.0 software by the neighbor-joining method.
